# IoT Device Security: Challenging “A Lightweight RFID Mutual Authentication Protocol Based on Physical Unclonable Function”

**DOI:** 10.3390/s18124444

**Published:** 2018-12-15

**Authors:** Ygal Bendavid, Nasour Bagheri, Masoumeh Safkhani, Samad Rostampour

**Affiliations:** 1 Department of Management and Technology, Université du Québec à Montréal (UQAM), Montreal, QC H2X 1L7, Canada; samad.rostampour@iauahvaz.ac.ir; 2 Electrical Engineering Department, Shahid Rajaee Teacher Training University, Tehran 16788-15811, Iran; NBagheri@sru.ac.ir; 3 School of Computer Science, Institute for Research in Fundamental Sciences (IPM), Tehran 19538-33511, Iran; 4 Computer Engineering Department, Shahid Rajaee Teacher Training University, Tehran 16788-15811, Iran; Safkhani@sru.ac.ir; 5 Department of Computer Engineering, Ahvaz Branch, Islamic Azad University, Ahvaz 61349-37333, Iran

**Keywords:** IoT, RFID, security, physical unclonable function, authentication protocol, desynchronization attack

## Abstract

With the exponential increase of Internet of things (IoT) connected devices, important security risks are raised as any device could be used as an attack channel. This preoccupation is particularly important with devices featuring limited processing power and memory capabilities for security purposes. In line with this idea, Xu et al. (2018) proposed a lightweight Radio Frequency Identification (RFID) mutual authentication protocol based on Physical Unclonable Function (PUF)—ensuring mutual tag-reader verification and preventing clone attacks. While Xu et al. claim that their security protocol is efficient to protect RFID systems, we found it still vulnerable to a desynchronization attack and to a secret disclosure attack. Hence, guidelines for the improvements to the protocol are also suggested, for instance by changing the structure of the messages to avoid trivial attacks. In addition, we provide an explicit protocol for which our formal and informal security analysis have found no weaknesses.

## 1. Introduction

The Internet of things (IoT) concept finds its roots in the early 1990s with the vision of ubiquitous computing [1] and the underlying idea that any object can be equipped with technology to become a computing device. This path to digital transformation is now possible as any object can communicate electronically and interact autonomously in real time, with its environment. In fact, the IoT landscape is rapidly changing with an increasing number of vertical applications moving “from experimentation to business scale” [2].

As the IoT market opens up multiple business opportunities, it also introduces a wealth of problems among which security issues usually top the list. This concern will keep increasing exponentially, as the worldwide number of connected devices is expected to jump by 12% on average annually, up to 125 billion connected devices in 2030 [3]. This will lead to important security issues as the ecosystem of connected devices is getting more complex and fragmented. Accordingly, experts anticipate that “by 2020, more than 25% of identified attacks in enterprises will involve the IoT, although the IoT will account for less than 10% of IT security budgets” [4]. The authors raise the fact that the IoT introduces new challenges (e.g., complexity in solution design architecture, implementation process and integration) as well as a wide range of new security risks “to the IoT devices themselves, their platforms and operating systems, their communications and even the systems to which they’re connected”. For instance, they suggest that any IoT device could be used as an attack channel.

Therefore, understanding how to build low-cost-high-security IoT devices becomes critical. This awareness is of a particular importance since IoT devices have become “so seamlessly integrated into everyday life, that often the end users are completely unaware of the presence of these devices around them” [5]. This is especially true for low-cost connecting devices in the IoT ecosystem, such as (battery-less) passive Radio-Frequency Identification (RFID) tags, that are powered by the interrogator’s radio waves, i.e., by transmitting their signal upon request, when interrogated by a reader. For instance, while passive Ultra High Frequency (UHF) tags are increasingly used in a variety of applications (e.g., identifying products and assets in logistic applications as well as participants in event management), these tags feature limited processing power and memory capabilities for security purposes (i.e., 16-bit cyclic-redundancy check (CRC)) [6].

In a report from the NIST (National Institute of Standards and Technology) [7], the authors present specific recommendations to address potential RFID security risks, including firewalls that separate RFID databases, encryption of radio signals, authentication of approved users, shielding of a reading zone to prevent unauthorized access, logging and time stamping to help in detecting security breaches, etc. Another way of securing RFID tags to support authentication applications is to create hardware security at the silicon level, by using a unique digital signature for RFID silicon chips based on how electrons flow through different paths of the chip, creating a silicon “fingerprint”. This approach, firstly introduced by Gassend et al. [8] at the Computer and Communication Security Conference in 2002, was put into practice in 2008 [9], when Verayo, an MIT spinout company, proposed “unclonable” HF tags which included small electronic circuits called PUFs (Physically Unclonable Functions). PUFs take advantage of variations in how silicon Integrated Circuits (ICs) are produced to create a unique digital signature for each chip, similar to biometrics measures, where the “identifiers” cannot be cloned. This allows a challenge–response authentication process that takes into account each tag unique identity based on its IC/transistor random electric properties.

While using chips “digital fingerprint” to build more secure tags, not all proposed PUFs are “unclonable”. For instance, in a previous paper entitled “A Lightweight RFID Mutual Authentication Protocol Based on Physical Unclonable Function”, Xu et al. [10] examined Kulseng et al.’s [11] proposal of a lightweight mutual verification protocol for RFID systems. Xu et al. found shortcomings in this protocol, making it vulnerable for a desynchronization attack. They hence proposed a security protocol for ensuring mutual tag-reader verification and preventing clone attacks. While the authors claim that their three-stage protocol (tag recognition, mutual verification and update) is efficient to protect RFID systems, we found that Xu et al.’s protocol is still vulnerable to a desynchronization attack and to a secret disclosure attack since the mutual verification process has flaws. These topics and arguments are addressed in this article.

The structure of this paper is as follows: the IoT and related security issues are raised in Section 2. RFID authentication protocols using PUF are discussed in Section 3, and in Section 4 Xu et al.’s protocol will be described. In Section 5, we will then evaluate the security level of the protocol and show its vulnerability. After that, we will present an improved protocol in Section 6 and finally we will conclude in Section 7.

## 2. The Internet of Things: Infrastructure and Security Issues

The International Telecommunication Union (ITU) defines the IoT as “a global infrastructure enabling advanced services by interconnecting physical and virtual things (i.e., living and non-living entities) based on existing and evolving interoperable information and communication technologies” [12]. The IoT, as a new technological paradigm that aims “to connect anything and anyone at any time and any place”, [13], is a vision becoming reality with burgeoning IoT enabled applications which open up to multiple business opportunities [14] and lead to new business models [15].

**Figure 1:**
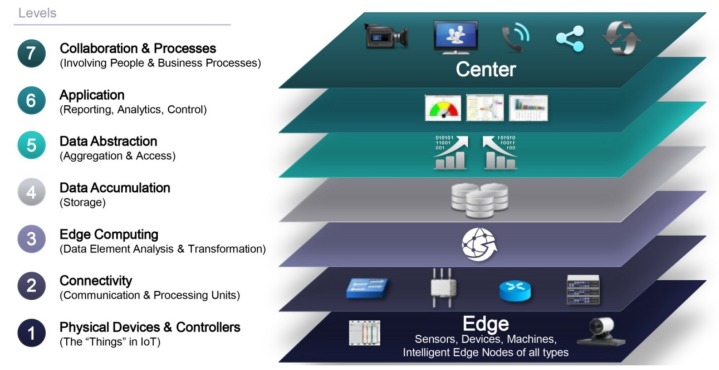
IoT reference model ([16]).

Figure 1 illustrates the IoT reference model ( [16]) comprised of seven levels, starting with Level 1: physical devices and controllers that might control multiple devices equipped with various technologies such as Radio Frequency Identification (RFID) tags, Bluetooth Low Energy (BLE) devices, Ultra Sound Identifications (USID), etc. Level 2 represents the communications and connectivity between devices and across networks. Level 3, called Edge (Fog) Computing, allows the conversion of network data flows into suitable information “for storage and higher level processing”. This happens at Level 4 (data accumulation), where event-based data is converted into query-based processing. Level 5 is then required to facilitate the integration with back end systems and “render data and its storage in ways that enable developing simpler, performance-enhanced applications”. It is at Level 6 (Application) that information interpretation takes place to automate or support decision-making in vertical markets’ applications. Level 7 hence represents empowered people and business processes using IoT enabled information to trigger action.

Since security issues can be raised for each level and for the movement of data between levels (e.g., physical cloning at Level 1, unauthorized access at Levels 2 and 3, data manipulation at Levels 4 and 5, and denial of service at Level 6), it goes without saying that security measures must pervade the entire model by (i) securing each device or system (ii) providing security for all processes at each level. Indeed, IoT security requires a multi-level approach.

## 3. RFID Authentication Protocols Using PUF

In this paper, we will focus on level of one of the IoT Reference Model, a level referred by Qi et al. [17] as “the sensing layer” or by Bendavid [18] as “the data capture layer”. At this level, one method to increase the security and to prohibit unauthorized access in IoT applications is to perform tag-reader mutual authentication protocol [19,20,21,22]. Various security techniques can be applied in order to transfer data securely such as cryptosystems. These cryptosystems are commonly divided into two groups: one-way and two-way [23]. In one-way systems, data is encrypted and there is no module for decrypting, such as Hash or PUF. In two-way systems, data is encrypted and decrypted by a cryptographic algorithm. Furthermore, within one-way systems, Hash, as a function, does not have the capability to detect physical attacks. On the other hand, PUF has an unclonable function that utilizes physical features to improve the security and resist against physical threats.

In recent years, many researchers have proposed more secure authentication protocols based on PUF technology [23,24]. For example, Sadeghi et al. presented an efficient privacy-preserving protocol based on rooted on PUF and claimed that their protocol was cost-efficient and provided an acceptable level of security and privacy [25]. However, despite Sadeghi et al.’s claim, Kardas et al. proved that the recommended protocol was vulnerable to the impersonation, as an attacker could trace the communication of a tag [26]. In addition, Kardas et al. analyzed another PUF-based authentication protocol that has been endorsed by Akgun et al. [27] and showed that the protocol was vulnerable and could not guarantee the security of the system [26]. Akgun et al. presented another authentication protocol by using PUF technology, but their protocol was still to be found insecure and could not provide forward secrecy support [28]. In order to solve the controversy surrounding these issues, Kardas et al. also presented another PUF-based protocol that was unfortunately not resistant against Denial of Service (DoS) attacks [29].

While most PUF based protocols were initially planned for an ideal environment with limited applicability in real situations, some researchers proposed authentication protocols for real and noisy environments [30,31,32,33]. For example, Ray et al. [34] released a PUF based protocol for object tracking in IoT application in real environments. In addition, Huth et al. proposed a protocol that can resist against noisy environment [33]. Nevertheless, their protocol needs an extra channel for transferring data and they use an exhaustive search in the database to retrieve the tag’s information. However, in their defense, most PUF protocols suffer the extra load of exhaustive search.

Recently, Kulseng et al. also disclosed a lightweight mutual verification protocol [11]. In order to implement a lightweight protocol, they utilized a PUF and Linear Feedback Shift Register (LFSR) instead of complex functions. However, while the proposed protocol was suitable for low-cost and large-scale systems, it could not comply with the security requirement of RFID systems. Xu et al. analyzed Kulseng et al.’s protocol and proved that it was vulnerable to the desynchronization attack and data confidentiality [10]. The authors presented a new PUF-based protocol and claimed that this improved protocol solved the security issues of the previous one and was robust against RFID threats. Before demonstrating its weaknesses, we will elaborate on Xu et al.’s protocol in more detail in Section 4.

## 4. Xu et al.’s Protocol

**Figure 2:**
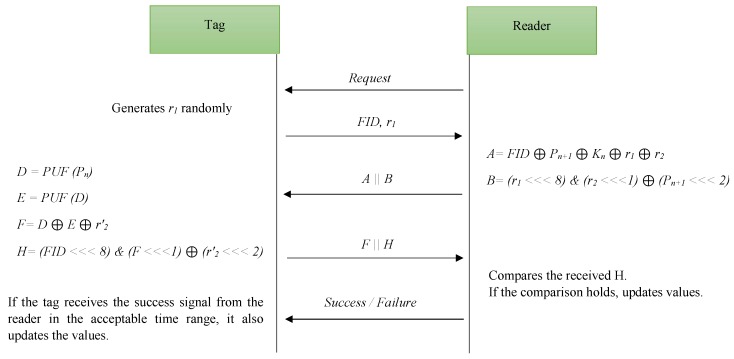
Time sequence diagram of Xu et al.’s protocol for single tag authentication.

In this section, we explain the PUF-based lightweight authentication protocol that has been proposed by Xu et al. The order of transferred messages is illustrated in Figure 2 and the used notations are listed in Table 1.

This protocol consists of four phases: Registration, Tag verification, Mutual verification and Update process. In the registration phase, the initial values of the parameters are stored in the tags and in the database. The database stores two sets of values for each tag: the current values ($FID^{new},P_{n}^{new},P_{n+1}^{new},K_{n}^{new}$) and the old values ($FID^{old},P_{n}^{old},P_{n+1}^{old},K_{n}^{old},ID$). If an authentication process is done completely and a tag updates its parameters successfully, the database uses the current values; otherwise, it will use the old values.

### 4.1. Tag Verification Phase

At the first of verification phase, the reader starts the process and sends a search request to the tag and receives the tag’s response as FID and $r_{1}$. The reader looks up FID in the new fields and old fields. Finally, if the appropriate FID is not found, the reader terminates the session; otherwise, it continues the process.

### 4.2. Mutual Verification Phase

In this phase, the reader generates $r_{2}$ randomly and computes *A* and *B* as:

(1) $$A=FID⊕P_{n+1}⊕K_{n}⊕r_{1}⊕r_{2},$$

(2) $$B=\left(r_{1}⋘8\right)\&\left(r_{2}⋘1\right)⊕\left(P_{n+1}⋘2\right).$$

It concatenates *A* and *B* and transfers the combination message (A||B) to the tag.

Upon receiving the message from the reader, the tag computes *D* and *E* as follows:

(3) $$D=PUF(P_{n}),$$

(4) E=PUF(D).

In addition, it calculates $r_{2}^{\prime }$ by the receiving messages to recalculate $B^{\prime }$ as:

(5) $$r_{2}^{\prime }=A⊕FID⊕D⊕K_{n}⊕r_{1},$$

(6) $$B^{\prime }=\left(r_{1}⋘8\right)\&\left(r_{2}^{\prime }⋘1\right)⊕\left(D⋘2\right).$$

The tag checks $B=B^{\prime }$. If the equation does not hold, it means that the reader is not valid and the session is terminated. Otherwise, the tag verifies the reader and continues the process.

Based on *D*, *E* and calculated $r_{2}^{\prime }$, the tag computes *F* and then *H* as:

(7) $$F=D⊕E⊕r_{2}^{\prime },$$

(8) $$H=\left(FID⋘8\right)\&\left(F⋘1\right)⊕\left(r_{2}^{\prime }⋘2\right).$$

It concatenates *F* and *H* as (F||H) and sends this message to the reader.

When the reader receives the tag’s response, by local parameters, it recalculates $E^{\prime }$, $F^{\prime }$ and $H^{\prime }$ as:

(9) $$E^{\prime }=F⊕r_{2}⊕P_{n+1},$$

(10) $$F^{\prime }=E^{\prime }⊕r_{2}⊕P_{n+1},$$

(11) $$H=\left(FID⋘8\right)\&\left(F^{\prime }⋘1\right)⊕\left(r_{2}⋘2\right).$$

Then, it checks $H=H^{\prime }$. If the equation does not hold, the tag is not valid and the reader aborts the session. Otherwise, it verifies the tag and continues the process.

### 4.3. Update Process Phase

After the verification phase, the variables should be updated. At first, the database updates its parameters, and then it sends a signal to the tag to inform it that the update process was done. If the tag receives the signal in the acceptable time range, it also updates its local parameters. Before updating in the database, the reader checks FID in the tag verification phase. If it has used $FID^{old}$, the reader does not need to update the parameters, but if $FID^{new}$ has been used, the reader updates the database as:

(12) $$P_{n}^{new}=P_{n+1}^{new},\;\;P_{n+1}^{new}=E^{\prime },$$

(13) $$K_{n}^{new}=\left(K_{n}^{new}⋘8\right)\&\left(r_{2}^{\prime }⋘1\right)⊕\left(r_{1}⋘2\right),$$

(14) $$FID^{new}=\left(FID^{new}⋘8\right)\&\left(r_{2}^{\prime }⋘1\right)⊕\left(r_{1}⋘2\right),$$

(15) $$FID^{old}=FID^{new},P_{n}^{old}=P_{n}^{new},P_{n+1}^{old}=P_{n+1}^{new},K_{n}^{old}=K_{n}^{new}.$$

If the tag receives a message (positive signal) from the reader in the acceptable time range, it also updates the parameters as:

(16) $$P_{n}=D,$$

(17) $$FID=\left(FID⋘8\right)\&\left(r_{2}^{\prime }⋘1\right)⊕\left(r_{1}⋘2\right),$$

(18) $$K_{n}=\left(K_{n}⋘8\right)\&\left(r_{2}^{\prime }⋘1\right)⊕\left(r_{1}⋘2\right).$$

## 5. Security Analysis of Xu et al.’s Protocol

While Xu et al. claimed that their security protocol is efficient to protect RFID systems, we found that their protocol is still at risk of being exposed to a desynchronization attack and to a secret disclosure attack. In this section, we will explain how these attacks can strongly jeopardize the security of the system.

### 5.1. Desynchronization Attack

The goal of a desynchronization attack is to change the integrity and synchronization of stored data in the tag and in the reader [35]. The main observation in the proposed attack mentioned in this section is the fact given A&B=C; any modification on a bit of *A* has no effect on the result *C* with the probability of $\frac{1}{2}$. More precisely, we assume that $B_{i}=0$, where $B_{i}$ denotes the ith bit of the string *B*, $A_{i}\&B_{i}=0$, for any value of $A_{i}\in \left\{0,1\right\}$. Following this observation, the attack procedure, which is a desynchronization attack when the adversary is modeled as a man in the middle, works as presented in the following steps:

1. On a legitimate session between the target tag *T* and the reader *R*, the reader starts the process and sends a search request to the tag.
2. Upon receiving the request, the tag generates $r_{1}$ randomly and transfers FID and $r_{1}$ to the reader.
3. The adversary intercepts the sent message from the tag to the reader and replaces $r_{1}$ with $r_{1}⊕0x1$.
4. When the reader receives FID, it utilizes it as a search key in the database and seeks to find an equal FID in the $FID^{new}$ field. We assume that the comparison holds, meaning that the tag belongs to the system and the reader uses $FID^{new},P_{n}^{new},P_{n+1}^{new},K_{n}^{new}$ for the next phase.
5. The reader generates $r_{2}$ randomly and computes *A* and *B* as:
  (19) $$A=FID⊕P_{n+1}⊕K_{n}⊕\left(r_{1}⊕0x1\right)⊕r_{2},$$
  (20) $$B=\left(\left(r_{1}⊕0x1\right)⋘8\right)\&\left(r_{2}⋘1\right)⊕\left(P_{n+1}⋘2\right).$$
6. It concatenates *A* and *B* and transfers the combination message (A||B) to the tag.
7. The adversary intercepts the sent message from the reader to the tag and replaces *A* with A⊕0x1.
8. Upon receiving the message from the reader, the tags computes *D* and *E* as follows:
  (21) $$D=PUF(P_{n}),$$
  (22) E=PUF(D).
9. In addition, it calculates $r_{2}^{\prime }$ by the receiving messages to recalculate $B^{\prime }$ as:
  (23) $$r_{2}^{\prime }=A⊕FID⊕D⊕K_{n}⊕r_{1},$$
  (24) $$B^{\prime }=\left(r_{1}⋘8\right)\&\left(r_{2}^{\prime }⋘1\right)⊕\left(D⋘2\right).$$
10. The tag checks $B=B^{\prime }$. If the equation does not hold, it means the reader is not valid and the session is terminated. Otherwise, the tag verifies the reader and continues the process.
11. Based on *D*, *E* and calculated $r_{2}^{\prime }$, the tag computes *F* and then *H* as:
  (25) $$F=D⊕E⊕r_{2}^{\prime },$$
  (26) $$H=\left(FID⋘8\right)\&\left(F⋘1\right)⊕\left(r_{2}^{\prime }⋘2\right).$$
12. It concatenates *F* and *H* as (F||H) and sends this message to the reader.
13. When the reader receives the tag’s response, by local parameters, it recalculates $E^{\prime }$, $F^{\prime }$ and $H^{\prime }$ as:
  (27) $$E^{\prime }=F⊕r_{2}⊕P_{n+1},$$
  (28) $$F^{\prime }=E^{\prime }⊕r_{2}⊕P_{n+1},$$
  (29) $$H=\left(FID⋘8\right)\&\left(F^{\prime }⋘1\right)⊕\left(r_{2}⋘2\right).$$
14. Then, it checks $H=H^{\prime }$. If it does not hold, the tag is not valid, and the reader aborts the session. Otherwise, it verifies the tag and continues the process.
15. Based on the assumption in Step 4, in the update process phase of the protocol verification phase, the database updates its parameters and then sends a signal to the tag to inform it that the update process is completed. The reader updates the database as follows:
  (30) $$P_{n}^{new}=P_{n+1}^{new}\;,\;P_{n+1}^{new}=E^{\prime },$$
  (31) $$K_{n}^{new}=\left(K_{n}^{new}⋘8\right)\&\left(r_{2}^{\prime }⋘1\right)⊕\left(\left(r_{1}⊕1\right)⋘2\right),$$
  (32) $$FID^{new}=\left(FID^{new}⋘8\right)\&\left(r_{2}^{\prime }⋘1\right)⊕\left(\left(r_{1}⊕1\right)⋘2\right),$$
  (33) $$FID^{old}=FID^{new},P_{n}^{old}=P_{n}^{new},P_{n+1}^{old}=P_{n+1}^{new},K_{n}^{old}=K_{n}^{new}.$$
16. When the tag receives the success signal from the reader in the acceptable time range, it also updates the parameters as:
  (34) $$P_{n}=D,$$
  (35) $$FID=\left(FID⋘8\right)\&\left(r_{2}^{\prime }⋘1\right)⊕\left(r_{1}⋘2\right),$$
  (36) $$K_{n}=\left(K_{n}⋘8\right)\&\left(r_{2}^{\prime }⋘1\right)⊕\left(r_{1}⋘2\right).$$

It is clear that the above attack succeeds if:

(37) $$\left(r_{1}⋘8\right)\&\left(r_{2}⋘1\right)⊕\left(P_{n+1}⋘2\right)=\left(\left(r_{1}⊕0x1\right)⋘8\right)\&\left(r_{2}⋘1\right)⊕\left(P_{n+1}⋘2\right).$$

In light of these findings, the probability of the above equality is $\frac{1}{2}$. Given that the only impact of $r_{1}$ is on the calculation of A∥B, if the above equality is satisfied, *T* and *R* authenticate each other successfully and the adversary is not recognized. However, the tag updates its values with $r_{1}$ while the reader uses $r_{1}⊕1$ in the update process. Hence, the tag’s records of secret parameters do not match the records of the reader and they will be desynchronized with the probability of almost $\frac{1}{2}$, while the complexity of the attack method is just intercepting two steps of a session of the protocol between *T* and *R*.

### 5.2. Secret Disclosure Attack

The main observation which is used in the proposed secret disclosure attack is the fact that $A_{i}\&0=0$ and $A_{i}\&1=A_{i}$, for any value of $A_{i}\in \left\{0,1\right\}$. Following this observation, the attack procedure, when the adversary is modeled as a man in the middle, works as follows:

1. On a legitimate session between the target tag *T* and the reader *R*, the reader starts the process and sends a search request to the tag.
2. Upon receiving the request, the tag generates $r_{1}$ randomly and transfers FID and $r_{1}$ to the reader.
3. The adversary intercepts the sent message from the tag to the reader and just replaces $r_{1}$ by 0 and stores FID.
4. When the reader receives FID, it utilizes it as a search key in the database and seeks to find an equal FID in the $FID^{new}$ field. We assume that the comparison holds, meaning that the tag belongs to the system and the reader uses $FID^{new},P_{n}^{new},P_{n+1}^{new},K_{n}^{new}$ for the next phase.
5. The reader generates $r_{2}$ randomly and computes *A* and *B* as:
  (38) $$A=FID⊕P_{n+1}⊕K_{n}⊕\left(0\right)⊕r_{2},$$
  (39) $$B=\left(0⋘8\right)\&\left(r_{2}⋘1\right)⊕\left(P_{n+1}⋘2\right)=P_{n+1}⋘2.$$
6. The adversary stores A∥B and blocks it, where $B=P_{n+1}⋘2$ and $P_{n+1}$ is the tag’s secret value and $A=FID⊕P_{n+1}⊕K_{n}⊕r_{2}$.
7. The adversary waits until the reader starts the process and sends a search request to a tag.
8. Upon receiving the request, the adversary sets $r_{1}^{\prime }=0x1\ldots 1$ and transfers the stored FID and $r_{1}^{\prime }$ to the reader.
9. When the reader receives FID, it utilizes it as a search key in the database and seeks to find an equal FID in the $FID^{new}$ field. Based on the assumption of Step 4, the comparison holds, meaning that the tag belongs to the system and the reader uses $FID^{new},P_{n}^{new},P_{n+1}^{new},K_{n}^{new}$ for the next phase.
10. The reader generates $r_{2}^{\prime }$ randomly and computes $A^{\prime }$ and $B^{\prime }$ as:
  (40) $$A^{\prime }=FID⊕P_{n+1}⊕K_{n}⊕\left(0x1\ldots 1\right)⊕r_{2}^{\prime },$$
  (41) $$B^{\prime }=\left(\left(0x1\ldots 1\right)⋘8\right)\&\left(r_{2}^{\prime }⋘1\right)⊕\left(P_{n+1}⋘2\right).$$
11. The adversary stores $A^{\prime }∥B^{\prime }$ and blocks it, where $B^{\prime }=\left(r_{2}^{\prime }⋘1\right)⊕\left(P_{n+1}⋘2\right)$. Given $P_{n+1}$ from Step 6, the adversary extracts $r_{2}^{\prime }$ as $r_{2}^{\prime }=\left(\left(B^{\prime }⊕\left(P_{n+1}⋘2\right)\right)⋙2\right)$ and $K_{n}$ as $K_{n}=A^{\prime }⊕FID⊕P_{n+1}⊕\left(0x1\ldots 1\right)⊕r_{2}$.

On the basis of these elements, the probability of success of the adversary to retrieve the tag’s secret value is 1, while the complexity of the attack method is just intercepting a step of the sessions of the protocol between *T* and *R* and eavesdropping the transferred values over a public channel. It should also be noted that, given $P_{n+1}$ and $K_{n}$, the adversary can impersonate the reader and communicate with the tag to extract the rest of the secret parameters as follows:

1. The adversary impersonates the legitimate reader and sends a search request to the target tag.
2. Upon receiving the request, the tag generates $r_{1}^{\prime \prime }$ randomly and transfers FID and $r_{1}^{\prime \prime }$ to the reader (adversary).
3. The adversary generates $r_{2}^{\prime \prime }$ randomly and computes $A^{\prime \prime }$ and $B^{\prime \prime }$ as follows and sends them to the tag:
  (42) $$A^{\prime \prime }=FID⊕P_{n+1}⊕K_{n}⊕r_{1}^{\prime \prime }⊕r_{2}^{\prime \prime },$$
  (43) $$B^{\prime \prime }=\left(r_{1}^{\prime \prime }⋘8\right)\&\left(r_{2}^{\prime \prime }⋘1\right)⊕\left(P_{n+1}⋘2\right).$$
4. Upon receiving the message, the tag computes $D=PUF(P_{n})$ and E=PUF(D), verifies the received $B^{\prime \prime }$ to validate the reader and continues the process.
5. Based on *D*, *E* and calculated $r_{2}^{\prime }$, the tag computes *F* and then *H* as:
  (44) $$F^{\prime \prime }=D⊕E⊕r_{2}^{\prime \prime },$$
  (45) $$H^{\prime \prime }=\left(FID⋘8\right)\&\left(F^{\prime \prime }⋘1\right)⊕\left(r_{2}^{\prime \prime }⋘2\right).$$
6. It concatenates $F^{\prime \prime }$ and $H^{\prime \prime }$ as $\left(F^{\prime \prime }||H^{\prime \prime }\right)$ and sends this message to the reader (adversary).
7. When the adversary receives the tag’s response, it extracts $E=D⊕F^{\prime \prime }⊕r_{2}^{\prime \prime }$, sends a signal to the tag to inform that the update process was done, and does the following updates in the stored information related to the target tag:
  (46) $$P_{n}^{new}=P_{n+1},\;\;P_{n+1}^{new}=E,$$
  (47) $$K_{n}^{new}=\left(K_{n}^{new}⋘8\right)\&\left(r_{2}^{\prime \prime }⋘1\right)⊕\left(\left(r_{1}^{\prime \prime }⊕1\right)⋘2\right),$$
  (48) $$FID^{new}=\left(FID^{new}⋘8\right)\&\left(r_{2}^{\prime \prime }⋘1\right)⊕\left(\left(r_{1}^{\prime \prime }⊕1\right)⋘2\right).$$
8. When the tag receives the success signal from the reader (adversary) in the acceptable time range, it also updates the parameters as:
  (49) $$P_{n}=D,$$
  (50) $$FID=\left(FID⋘8\right)\&\left(r_{2}^{\prime \prime }⋘1\right)⊕\left(r_{1}^{\prime \prime }⋘2\right),$$
  (51) $$K_{n}=\left(K_{n}⋘8\right)\&\left(r_{2}^{\prime \prime }⋘1\right)⊕\left(r_{1}^{\prime \prime }⋘2\right).$$

From now on, the adversary is in possession of all the tags secrets and has also desynchronized the tag and the reader. Hence, it is only the adversary who can communicate with the victim tag. The probability of success of the attack is 1 and the complexity of the attack method is negligible. One may argue that, in order to patch the given secret discourse attack, the reader shall refuse trivial values as $r_{1}$, e.g., it should reject any value which does not sound like it is random such as $r_{1}=0\ldots 0$ and $r_{1}=1\ldots 1$. However, this approach does not rule out the projected attack, although it can slightly increase the complexity of the attack. In this case, the scenario of an attack could be as follows:

1. In a legitimate session between the target tag *T* and the reader *R*, the reader starts the process and sends a search request to the tag.
2. Upon receiving the request, the tag generates $r_{1}$ randomly and transfers FID and $r_{1}$ to the reader.
3. The adversary intercepts the sent message from the tag to the reader and just replaces $r_{1}$ by 0 and stores FID.
4. When the reader receives FID, it utilizes it as a search key in the database and seeks to find an equal FID in the $FID^{new}$ field. We assume that the comparison holds, meaning that the tag belongs to the system and the reader uses $FID^{new},P_{n}^{new},P_{n+1}^{new},K_{n}^{new}$ for the next phase.
5. The reader generates $r_{2}$ randomly and computes *A* and *B* as:
  (52) $$A=FID⊕P_{n+1}⊕K_{n}⊕r_{1}⊕r_{2},$$
  (53) $$B=\left(r_{1}⋘8\right)\&\left(r_{2}⋘1\right)⊕\left(P_{n+1}⋘2\right).$$
6. The adversary stores A∥B and blocks it, where, for any ${\left(r_{1}\right)}_{i}=0$, we have $B_{i+6}={\left(P_{n+1}\right)}_{i+6}$ and $P_{n+1}$ is the tag’s secret value and $A=FID⊕P_{n+1}⊕K_{n}⊕r_{2}$.
7. The adversary waits until the reader starts another session of the protocol and sends a search request to a tag.
8. Upon receiving the request, the adversary sets $r_{1}^{\prime }=r_{1}⊕0x1\ldots 1$ and transfers the stored FID and $r_{1}^{\prime }$ to the reader.
9. When the reader receives FID, it utilizes it as a search key in the database and seeks to find an equal FID in the $FID^{new}$ field. We again assume that the comparison holds, meaning that the tag belongs to the system and the reader uses $FID^{new},P_{n}^{new},P_{n+1}^{new},K_{n}^{new}$ for the next phase.
10. The reader generates $r_{2}^{\prime }$ randomly and computes $A^{\prime }$ and $B^{\prime }$ as:
  (54) $$A^{\prime }=FID⊕P_{n+1}⊕K_{n}⊕r_{1}^{\prime }⊕r_{2}^{\prime },$$
  (55) $$B^{\prime }=\left(r_{1}^{\prime }⋘8\right)\&\left(r_{2}^{\prime }⋘1\right)⊕\left(P_{n+1}⋘2\right).$$
11. The adversary stores $A^{\prime }∥B^{\prime }$ and blocks it, where, for any ${\left(r_{1}^{\prime }\right)}_{i}=0$$B_{i+6}={\left(P_{n+1}\right)}_{i+6}$. Combining these bits of $P_{n+1}$ with the bits retrieved in Step 6, the adversary has a whole $P_{n+1}$.
12. The adversary waits until the reader starts the next process and sends a search request to a tag.
13. Upon receiving the request, the adversary generates a random value as $r_{1}$, transfers $r_{1}$ and the stored FID to the reader.
14. When the reader receives FID, it utilizes it as a search key in the database and seeks to find an equal FID in the $FID^{new}$ field. Based on the assumption of Step 4, the comparison holds, meaning that the tag belongs to the system and the reader uses $FID^{new},P_{n}^{new},P_{n+1}^{new},K_{n}^{new}$ for the next phase.
15. The reader generates $r_{2}$ randomly and computes *A* and *B* as:
  (56) $$A=FID⊕P_{n+1}⊕K_{n}⊕r_{1}⊕r_{2},$$
  (57) $$B=\left(\left(0x1\ldots 1\right)⋘8\right)\&\left(r_{2}⋘1\right)⊕\left(P_{n+1}⋘2\right).$$
16. The adversary stores A∥B and blocks it, where, for any ${\left(r_{1}\right)}_{i}=1,$ we have $B_{i+6}={\left(P_{n+1}\right)}_{i+6}⊕r_{1}⊕{\left(r_{2}\right)}_{i+7}$ and $A_{i+7}={\left(FID⊕P_{n+1}⊕K_{n}⊕r_{2}\right)}_{i+7}$. Given that the adversary has FID, $P_{n+1}$, $r_{1}$ and ${\left(r_{2}\right)}_{i+7}$, it can extract ${\left(K_{n}\right)}_{j}$ for any *j* that ${\left(r_{1}\right)}_{j-7}=1$.
17. The adversary waits until the reader starts another session of the protocol and sends a search request to a tag.
18. Upon receiving the request, the adversary sets $r_{1}^{\prime }=r_{1}⊕0x1\ldots 1$ and transfers the stored FID and $r_{1}^{\prime }$ to the reader.
19. When the reader receives FID, it utilizes it as a search key in the database and seeks to find an equal FID in the $FID^{new}$ field. We again assume that the comparison holds, meaning that the tag belongs to the system and the reader uses $FID^{new},P_{n}^{new},P_{n+1}^{new},K_{n}^{new}$ for the next phase.
20. The reader generates $r_{2}^{\prime }$ randomly and computes $A^{\prime }$ and $B^{\prime }$ as:
  (58) $$A^{\prime }=FID⊕P_{n+1}⊕K_{n}⊕r_{1}^{\prime }⊕r_{2}^{\prime },$$
  (59) $$B^{\prime }=\left(r_{1}^{\prime }⋘8\right)\&\left(r_{2}^{\prime }⋘1\right)⊕\left(P_{n+1}⋘2\right).$$
21. ${\left(r_{1}\right)}_{i}=1$, we have $B_{i+6}^{\prime }={\left(P_{n+1}\right)}_{i+6}⊕{\left(r_{2}^{\prime }\right)}_{i+7}$ and $A_{i+7}^{\prime }={\left(FID⊕P_{n+1}⊕K_{n}⊕r_{1}^{\prime }⊕r_{2}^{\prime }\right)}_{i+7}$. Given that the adversary has FID, $P_{n+1}$, $r_{1}^{\prime }$ and ${\left(r_{2}^{\prime }\right)}_{i+7}$, it can extract ${\left(K_{n}\right)}_{j}$ for any *j* that ${\left(r_{1}^{\prime }\right)}_{j-7}=1$. Combining these bits of $K_{n}$ with the bits retrieved in Step 16, the adversary has a whole $K_{n}$.

It is clear that, in the given attack, the probability of success of the adversary to retrieve the tag’s secret value is 1, while the complexity of the attack method is just intercepting/eavesdropping four sessions of the protocol between *T* and *R*. Even if the reader stores the history of all transferred random values between the tag and the reader, it is possible to adapt the given attack to deceive the reader and extract the secret parameters, although we may require a few extra number of sessions. It should be noted that we did not use the tag responses, i.e., *F* and *H*, in the proposed secret disclosure attacks. For example, in Xu et al.’s protocol, FID and *F* are also transferred over a public channel and:

(60) $$F=D⊕E⊕r_{2},$$

(61) $$H=\left(FID⋘8\right)\&\left(F⋘1\right)⊕\left(r_{2}⋘2\right).$$

Hence, the adversary can easily extract $r_{2}$ from the available information. Given $r_{2}$ and $r_{1}$ and A∥B, where:

(62) $$A=FID⊕P_{n+1}⊕K_{n}⊕r_{1}⊕r_{2},$$

and

(63) $$B=\left(r_{1}⋘8\right)\&\left(r_{2}⋘1\right)⊕\left(P_{n+1}⋘2\right).$$

The adversary can easily extract $P_{n+1}$ and $K_{n}$. Given $r_{2}$ and $P_{n+1}=D$ and *F*, the adversary can extract *E*. Hence, the adversary extracts all secret parameters passively. The probability of success of this attack is also 1. However, it necessarily requires eavesdropping *F* and *H*.

## 6. Improved Protocol

To avoid trivial attacks, it is possible to design a new protocol based on Xu et al.’s protocol by changing the structure of the messages programmed. For instance, any transferred information should be masked. Therefore, it is better to transfer $r_{1}⊕P_{n}$ instead of the plain $r_{1}$ or use ID to generate the new TID rather than the TID itself, which is known by the adversary. Furthermore, considering the fact that the output of AND operation is ‘0’ with the probability of $\frac{3}{4}$, one should avoid it and try to use XOR which is a balanced bitwise operation. However, all things considered, the history of many easily broken ultra-lightweight protocols in literature shows that even such an improved protocol may not be that secure, not to mention that we use enough nonlinear and complicated functions, instead of just a few bitwise operations. Under the circumstances, it could be better to use available well-known lightweight primitives such as SIMON [36], Skinny [37], NORX [38] and SPONGENT [39]. For example, it is possible to implement SPONGNET in an area equal to 738 NAND gate [40]. Based on this observation, we propose in this section an improved authentication protocol apart from PUF, which uses a cryptographic hash function as the core of security. It should be noted many lightweight hash functions have already been introduced in literature, e.g., PHOTON [41], Quark [42] and SPONGENT [39]. A comprehensive comparison of those primitives can be found at CryptoLUX [40].

### 6.1. The Proposed Protocol

**Figure 3:**
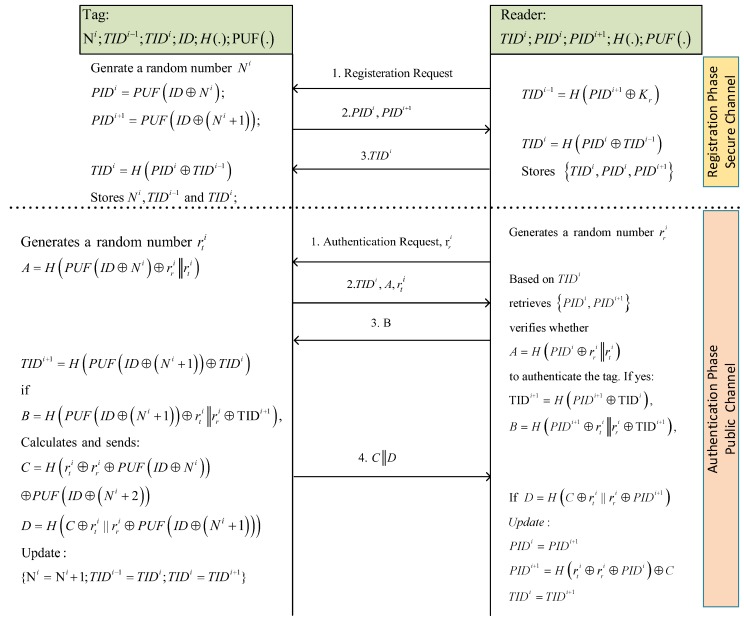
The improved PUF-based protocol.

The proposed protocol, as it is depicted in Figure 3, includes two phases: the registration phase over a secure channel and the authentication phase over a public channel.

**Registration Phase:**

In this phase, the tag $T^{i}$ is registered into the reader as follows:

1. The reader sends a registration request command to the tag.
2. The tag generates a random number $N^{i}$, calculates $PID^{i}$ and $PID^{i+1}$ as follows and sends them to the reader:
  $$\begin{array}{c} PID^{i}=PUF\left(ID⊕N^{i}\right),\\ PID^{i+1}=PUF\left(ID⊕\left(N^{i}+1\right)\right). \end{array}$$
3. The reader calculates a temporary identifier $TID^{i-1}=H\left(PID^{i+1}⊕K_{r}\right)$ for the tag and sends to it, where $K_{r}$ is the secret parameter of the reader.
4. It also updates $TID^{i}$ as $TID^{i}=H\left(PID^{i}⊕TID^{i-1}\right)$ and creates a record in its database for this tag, including $\left\{TID^{i},PID^{i},PID^{i+1}\right\}$.
5. Given $TID^{i-1}$, the tag calculates $TID^{i}=H\left(PID^{i}⊕TID^{i-1}\right)$. It stores $\left\{N^{i},TID^{i-1},TID^{i}\right\}$.

**Authentication Phase:**

In this phase, the tag $T^{i}$ and the reader *R* are mutually authenticated as follows:

1. The reader sends an authentication request command along a random number $r_{r}^{i}$ to the tag.
2. The tag generates a random number $r_{t}^{i}$, calculates $A=H\left(PUF\left(ID⊕N^{i}\right)⊕r_{r}^{i}∥r_{t}^{i}\right)$ and sends tuple $\left(A,TID^{i},r_{t}^{i}\right)$ to the reader.
3. Given $TID^{i}$, the reader finds the related record in its database, i.e., $\left\{TID^{i},PID^{i},PID^{i+1}\right\}$. Then, the reader verifies whether $A\overset{?}{=}H\left(PID^{i}⊕r_{r}^{i}∥r_{t}^{i}\right)$ to authenticate the tag. If the tag is authenticated, then the reader calculates $TID^{i+1}=H\left(PID^{i+1}⊕{TID}^{i}\right)$ and $B=H\left(PID^{i+1}⊕r_{t}^{i}∥r_{r}^{i}⊕TID^{i+1}\right)$. Next, it sends *B* to the tag.
4. The tag also calculates $TID^{i+1}=H\left(PUF\left(ID⊕(N^{i}+1)\right)⊕TID^{i}\right)$ and verifies whether $B\overset{?}{=}H\left(PUF\left(ID⊕(N^{i}+1)\right)⊕r_{t}^{i}∥r_{r}^{i}⊕TID^{i+1}\right)$, to authenticate the reader. If the reader is certified as valid, the tag does the following calculations:
  $$\begin{array}{c} C=H\left(r_{t}^{i}⊕r_{r}^{i}⊕PUF\left(ID⊕N^{i}\right)\right)⊕PUF\left(ID⊕\left(N^{i}+2\right)\right),\\ D=H\left(C⊕r_{t}^{i}∥r_{r}^{i}⊕PUF\left(ID⊕(N^{i}+1)\right)\right). \end{array}$$
5. Next, the tag sends (C,D) to the reader and updates its records as $\left\{N^{i}=N^{i}+1;\;TID^{i-1}=TID^{i};TID^{i}=TID^{i+1}\right\}$.
6. Once the reader received (C,D), it verifies whether $D\overset{?}{=}H\left(C⊕r_{t}^{i}∥r_{r}^{i}⊕PID^{i+1}\right)$, to authenticate the tag and the received data. If it is authenticated, then the reader updates its records as follows:
  $$\begin{array}{c} PID^{i}=PID^{i+1},\\ PID^{i+1}=H\left(r_{t}^{i}⊕r_{r}^{i}⊕PID^{i}\right)⊕C,\\ TID^{i}=TID^{i+1}. \end{array}$$
7. If the tag has not been authenticated based on $N^{i}$ and $TID^{i}$, it assumes that the last session was not finished successfully and the tag tries to be authenticated based on $N^{i}-1$ and $TID^{i-1}$.

### 6.2. Security Evaluation of the Proposed Protocol

In this section, we evaluate the security of the proposed protocol informally and formally. For this purpose, we considered an active adversary, which is able to eavesdrop on any transferred messages over the public channel at different stages of the procedure, e.g., authentication phase of the proposed protocol, modify/block messages or initiate a session to impersonate the reader/tag. However, we specify that the adversary cannot influence or access the transferred messages over a secured channel, e.g., registration phase of the proposed protocol. In addition, we assume that the embedded PUF behaves randomly from a tag to a tag and on different inputs, i.e., $Pr\left(PUF^{i}\left(x_{1}\right)=PUF^{i}\left(x_{2}\right)\right)=2^{-n}$ and $Pr\left(PUF^{i}\left(x_{1}\right)=PUF^{j}\left(x_{1}\right)\right)=2^{-n}$, where $x_{1}\neq x_{2}$, *n* is the output length of $PUF^{i}$ and $PUF^{i}$ is the PUF which is integrated in the tag $T^{i}$.

#### 6.2.1. Informal Analysis

**Traceability Attack:** To trace a tag/reader and to compromise the protocol’s anonymity, the adversary should be able to link transferred messages between the tag and the reader to their identity or to the previous transferred messages. On the other hand, on each run of the protocol, the transferred messages include $r_{r}^{i},$$r_{t}^{i}$, $TID^{i}$, $A=H\left(PID^{i}⊕r_{r}^{i}∥r_{t}^{i}\right)$, $B=H\left(PID^{i+1}⊕r_{t}^{i}∥r_{r}^{i}⊕TID^{i+1}\right)$, $C=H\left(r_{t}^{i}⊕r_{r}^{i}⊕PUF\left(ID⊕N^{i}\right)\right)⊕PUF\left(ID⊕\left(N^{i}+2\right)\right)$ and $D=H\left(C⊕r_{t}^{i}∥r_{r}^{i}⊕PUF\left(ID⊕(N^{i}+1)\right)\right)$, where $TID^{i+1}=H\left(PID^{i+1}⊕{TID}^{i}\right)$. $r_{r}^{i}$ and $r_{t}^{i}$ are fresh random values generated in each session and they cannot be used to trace the tag/reader. $TID^{i}$ is the pseudo identity of the tag which is updated at the end of each successful run of the protocol. Moreover, the tag keeps its record up to the next successful run of the protocol. Although the record can be used to trace the tag even after one successful run of the protocol, it is impossible to employ it later.

More precisely, an adversary who eavesdropped on a session of the protocol between the tag and the reader and who missed two consecutive successful runs of the protocol after that will not be able to use the eavesdropped value of $TID^{i}$ to trace the target tag. It should be noted that it is possible to fix this problem by masking the transferred pseudo-identity, for example as $H(TID^{i+1}⊕r_{t}^{i})$. However, in doing so, the reader needs to search through its entire database to find the tag that compromises the protocol’s scalability. Other solutions, such as encrypting TID by the public key of the reader, are also possible, but it increases the computational complexity of the protocol. The rest of the messages are randomized by random values contributed by both the tag and the reader and a secure hash function. Hence, even an adversary who is impersonating the reader or the tag cannot control the random numbers generated by the other party and cannot predict the transferred messages from one session to another session.

**Secret Disclosure Attack:** The main secret parameters that are shared with the reader are $PID^{i}$ and $PID^{i+1}$. They are used in generating different messages that are transferred between the tag and the reader, i.e., *A*, *B*, *C* and *D*. However, they are mainly used as the input of a hash function that is not possible to invert efficiently. The only way to achieve such an inversion should be to use $C=H\left(r_{t}^{i}⊕r_{r}^{i}⊕PID^{i}\right)⊕PID^{i+2}$ to determine $PID^{i+2}$. However, it is randomized by $H\left(r_{t}^{i}⊕r_{r}^{i}⊕PID^{i}\right)$ for which the adversary has no knowledge and cannot control. Hence, the adversary cannot efficiently determine $PID^{i}$ and $PID^{i+1}$.

**Impersonation Attack:** Given the fact that the adversary cannot determine $PID^{i}$ and $PID^{i+1}$, to impersonate a tag to the reader, the adversary should at least generate a valid *A*. However, to produce it, the adversary ought to appreciate the knowledge of $PID^{i}$, since the previously eavesdropped messages cannot be used also due to the fresh $r_{r}^{i}$ and $r_{t}^{i}$ that are generated in each new session, the adversary cannot do this attack efficiently. A similar argument is also valid and relevant for the reader to the tag impersonation attack.

**Replay Attack:** Considering the fact that all messages that are critical to authenticate the tag by the reader or the reader by the tag are randomized by both parties, it is not possible to apply a replay attack against the proposed protocol.

**Tag Cloning Attack:** Assuming that the used PUF behaves randomly, it is impossible to clone a tag even when the adversary compromises the tag to access $TID^{i}$, $TID^{i-1}$ and $N^{i}$. The reason for this comes from the fact that it is not possible to implement identical PUF in different tags.

**Desynchronization Attack:** To desynchronize the tag and the reader, the reader should be able to impersonate the reader to the tag or the tag to the reader, which is not possible. Another approach would be by blocking the last message sent from the tag to the reader. In this way, the tag has updated its records but not the reader. However, given that the tag also keeps a record of the old data; in this case, it can be synchronized based on them.

**Forward/Backward Security:** Given that the tag and the reader share two parameters $PID^{i}$ and $PID^{i+1}$ and since, in each round, only one of them is changed while the tag keeps a history of old parameters, we define our forward security model as the case when the adversary has all parameters of session *x*, misses two successful sessions and aims to recover parameters in the ${\left(x+2\right)}^{th}$ session. It is clear that, in two consecutive sessions, both $PID^{i}$ and $PID^{i+1}$ are changed through PUF randomly, and, without them, the adversary has no advantage over a blind adversary. Hence, the adversary will not be able to determine $PID^{i}$ and $PID^{i+1}$. A similar reasoning applies to the backward security of the protocol.

#### 6.2.2. Formal Verification Using a Scyther Tool

Scyther [43] is a widely accepted tool to evaluate the security correctness of a protocol. It provides a graphical user interface to facilitate the analysis of complex attack scenarios on the target protocol. We applied the Scyther tool to verify whether our security assertions for the proposed protocol holds or not. We defined two identities over the protocol, i.e., *tag* and *reader* in Scyther. Then, we implemented each role with its corresponding task, followed by the implementations as represented in Appendix A. It is worth noting that we fixed the number of protocol runs 100 times and also fixed the search pruning to “Find all attacks“ and the matching type of Scyther tool to “Find all type flaws”.

We made our *Secret* claims on every message that was sent and received on both ends. According to Table 2, the Scyther tool could not find any attack within bounds, thus establishing that our security allegations were founded.

In terms of a tag’s weight and hardware complexity of the improved protocol, the protocol could belong to lightweight protocols. Accordingly, because PUF and hash functions have been used in the design of our proposed protocol, we can control the number of gates in the range of lightweight tags. Since a tag with less than 3000 GE [44] is lightweight, by utilizing a SPONGNET hash function with around 800 GE and some bitwise functions such as XOR, the total number of gates is less than 3000 and the proposed tag can satisfy the limitation of lightweight protocols. In addition, in terms of the security level, we compared the improved protocol with some other protocols in Table 3. The results show that our protocol could address the security issues of other protocols and is resistant against IoT attacks.

## 7. Conclusions

With the exponential increase of IoT connected devices, the importance of security risks is raised by the academic and professional community as any connected object becomes a computing device and a potential target for an attack. In developing strategies to identify and prevent intrusions and threats, which are more likely to increase, researchers around the word are working on securing all the layers of the IoT infrastructure. Along these trends, since the commercial introduction by Verayo in the early 2008 of the first silicon chips equipped with PUF, the market has seen some growth with other companies (e.g., Intrinsic ID, Quantum Trace, Invia) now developing in this marketplace. While there are no doubts that PUF constitutes a very promising avenue to ensure device security, caution should always be brought to constantly evolving security protocols. In line with this issue, Xu et al. proposed a lightweight RFID mutual authentication protocol based on PUF, for ensuring mutual tag-reader verification and preventing clone attacks. While the authors claimed that their protocol was efficient to protect RFID systems, we found it to still be vulnerable and presented how a desynchronization attack and several secret disclosure attacks could be performed on Xu et al.’s protocol. Finally, we discussed why it is better to try to employ secure lightweight encryption functions in designing secure protocols rather than ad hoc designs, by proposing a secure hash based protocol.

## Figures and Tables

**Table 1:** The notations used in the Xu et al.’s protocol.

Notations	
FID	Fake tag ID
PUF()	Physical Unclonable Function
ID	The tag’s ID number
TID	Pseudo ID of the tag
$P_{n}$	The tag’s secret value
$K_{n}$	The shared secret value of the tag and the reader
⋘	The left rotation operator
&	The AND operator
||	The concatenation operator
$r_{i}$	A random number
⊕	XOR function
$X_{i}$	Denotes the ith bit of the string X, where $X_{0}$ is the most right bit

**Table 2:** The proposed protocol verification results using the Scyther tool.

Claim				Status	Comments
improved	R	improved,R1	Secret ID	OK	No attacks within bounds.
		improved,R2	Niagree	OK	No attacks within bounds.
		improved,R3	Nisynch	OK	No attacks within bounds.
		improved,R4	Alive	OK	No attacks within bounds.
		improved,R5	Weakagree	OK	No attacks within bounds.
	T	improved,T1	Secret PIDi	OK	No attacks within bounds.
		improved,T2	Secret PIDip1	OK	No attacks within bounds.
		improved,T3	Niagree	OK	No attacks within bounds.
		improved,T4	Nisynch	OK	No attacks within bounds.
		improved,T5	Alive	OK	No attacks within bounds.
		improved,T6	Weakagree	OK	No attacks within bounds.

**Table 3:** The security comparison of the improved protocol to other protocols against IoT attacks.

Protocols	Impersonation	Traceability	Disclosure	Desynchronization
Sadeghi et al. [25]	×	×	✓	✓
Aysu et al. [32]	✓	×	×	✓
Van Herrewege et al. [30]	✓	✓	×	✓
Kulseng et al. [11]	✓	✓	×	×
Xu et al. [10]	✓	✓	×	×
Improved Protocol	✓	✓	✓	✓

✓: Resistant    ×: Non-resistant.

## Appendix A. Implementation of the Proposed Protocol in the Scyther Tool

hashfunction  h;

const xor:Function;

const con:Function;

const add:Function;

const puf:Function;

secret ID;

secret Ni;

secret TIDi;

secret PIDi;

secret PIDip1;

macro TIDip1=h(xor(PIDip1,TIDi));

macro A=h(con(xor(puf(xor(ID,Ni)),rri),rti));

macro B=h(con(xor(PIDip1,rti),xor(rri,TIDip1)));

macro C=xor(h(xor(rti,rri,puf(xor(ID,Ni)))),puf(xor(ID,add(Ni,2))));

macro D=h(con(xor(C, rti),xor(rri,puf(xor(ID,add(Ni,1))))));

protocol improved(R,T){

role R{

secret Ni;

secret TIDi;

secret PIDi;

secret PIDip1;

secret ID;

fresh rri;

var rti;

send_1(R,T,rri);

recv_2(T,R,A,TIDi,rti);

send_3(R,T,B);

recv_4(T,R,con(C,D));

claim(R,Secret,ID);

claim(R,Niagree);

claim(R,Nisynch);

claim(R,Alive);

claim(R,Weakagree);

}

role T{

fresh rti;

var rri;

secret Ni;

secret TIDi;

secret PIDi;

secret ID;

secret PIDip1;

recv_1(R,T,rri);

send_2(T,R,A,TIDi,rti);

recv_3(R,T,B);

send_4(T,R,con(C,D));

claim(T,Secret,PIDi);

claim(T,Secret,PIDip1);

claim(T,Niagree);

claim(T,Nisynch);

claim(T,Alive);

claim(T,Weakagree);

}}
